# 

**Published:** 1906-12-08

**Authors:** 


### BOOKS RECEIVED.

SIMPKIN, MARSHALL AND CO.

"For the Quiet Hour."

A. and C. BLACK.

"Who's Who," 1907.

"Who's Who Year-book," 1907.

F. A. DAVIS CO., PHILADELPHIA.

"Genito-Urinary Diseases and Syphilis," second edition. By H. M. Morton, M.D.

HARRISON AND SONS.

"Glimpses of American Surgery in 1906." By C. Hamilton Whiteford, M.R.C.S.

HADDEN, BEST AND CO.

"Hadden's Pocket Vocabulary of Medical Terms." Fourth edition. By Hy. Payne, M.D.

HENRY KIMPTON.

"Infant Education." By Eric Pritchard, M.D.

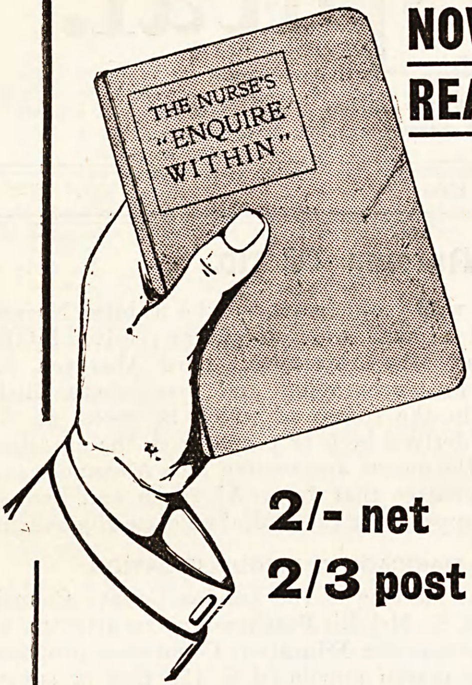

NOW READY.

2/- net
2/3 post free

You have often Needed a Book

That would aid you in emergency. That would help you to recollect some point you had forgotten or about which you had a doubt.

That would prove of assistance in difficulty.

That would convey information clearly and concisely on Diseases; their Symptoms and Nursing Treatment.

### The Nurse's "Enquire Within"

is a Pocket Encyclopædia of valuable nursing information alphabetically arranged, and

Fills a Want that has long been felt by Nurses everywhere. It is bound in limp cloth boards with rounded corners, and its size and weight will admit of its being carried conveniently in the apron pocket without injury thereto.

*The Book is obtainable of all Booksellers, or of*

The Scientific Press, Limited,

28/29 SOUTHAMPTON STREET,
STRAND, LONDON, W.C.

### Two Indispensable Handbooks for Nurses

NEW AND REVISED EDITION.

SURGICAL INSTRUMENTS AND APPLIANCES.

1/6 net, 1/8 post free.

An Illustrated and Classified List, with Explanatory Notes.

CONTAINING A NEW CHAPTER ON THE CARE AND STERILIZATION OF SURGICAL INSTRUMENTS.

BY

HAROLD BURROWS, M.B.(Lond.), B.S., F.R.C.S.

The NURSES' PRONOUNCING DICTIONARY OF MEDICAL TERMS AND NURSING TREATMENT.

2/- post free.

COMPILED BY

HONNOR MORTEN.

NEW EDITION.

Revised and Edited by MARY I. BURDETT.

Write for Catalogue of Nursing Manuals, which will be sent post free on application.

The Scientific Press, Ltd.,

*Publishers of Nursing Text-Books, Charts, and Case-Books of all Descriptions,*

28 & 29 SOUTHAMPTON STREET,
STRAND, LONDON, W.C.

